# Hereditary transthyretin amyloidosis in peripheral neuropathy: prevalence, clinical features, and neurology-based screening in non-endemic Japan

**DOI:** 10.3389/fneur.2026.1955913

**Published:** 2026-10-22

**Authors:** Yurika Okuma, Kenji Isahaya, Sachiko Ohori, Satomi Mitsuhashi, Tomoo Sato, Yoshihisa Yamano

**Affiliations:** 1 Department of Neurology, St. Marianna University School of Medicine, Kawasaki, Japan; 2 Department of Laboratory Medicine, St. Marianna University School of Medicine, Kawasaki, Japan; 3 Department of Rare Diseases Research, Institute of Medical Science, St. Marianna University School of Medicine, Kawasaki, Japan

**Keywords:** ATTRv, electrocardiography, genetic screening, hereditary transthyretin amyloidosis, Japan, neurology, peripheral neuropathy

## Abstract

**Introduction:**

Hereditary transthyretin amyloidosis (ATTRv) is a progressive, potentially fatal disease caused by pathogenic *TTR* variants. Although increasingly recognized as a cardiac disorder, ATTRv may initially present with peripheral neuropathy. Prospective data on the prevalence and clinical features of ATTRv among patients attending neurology clinics in non-endemic regions of Japan remain limited.

**Methods:**

We prospectively enrolled 130 consecutive patients evaluated for suspected peripheral neuropathy at a neurology clinic in a non-endemic region of Japan between 2020 and 2025. The entire *TTR* coding region was analyzed by bidirectional Sanger sequencing of genomic DNA obtained from peripheral blood. Clinical, neurological, and cardiac findings were evaluated.

**Results:**

Pathogenic *TTR* variants were identified in three patients (3/130, 2.31%; exact 95% CI, 0.5–6.6%): ATTRV122I [p.(Val142Ile)], ATTRI127V [p.(Ile147Val)], and ATTRV30M [p.(Val50Met)]. Two of the three variants were non-V30M variants unrelated to the Japanese endemic foci. All three patients had late-onset disease without a family history, with neurological manifestations ranging from established axonal or mixed polyneuropathy to early, mild involvement. Cardiac amyloid involvement was demonstrated in all three patients, including one with a structurally normal echocardiogram. A low-voltage electrocardiogram (ECG) was observed in all three ATTRv patients (3/3, 100%) compared with 5 of 95 (5.3%) variant-negative patients with available ECG data.

**Discussion:**

ATTRv was identified in 2.31% of patients evaluated for suspected peripheral neuropathy in a non-endemic Japanese neurology clinic, a frequency comparable to that reported in European cohorts. These findings highlight the importance of considering ATTRv in patients with unexplained progressive neuropathy, even without a family history or residence in an endemic region, and support early *TTR* genetic testing when clinical red flags are present. The observation of low-voltage ECGs in all three ATTRv patients suggests that ECG may provide a complementary clue to occult cardiac involvement. However, this finding is preliminary and hypothesis-generating and requires validation in larger, independent prospective cohorts.

## 1 Introduction

Amyloidosis comprises a group of disorders characterized by extracellular deposition of insoluble, misfolded proteins (amyloid) that progressively impair the affected organs. Transthyretin amyloidosis (ATTR) is related to transthyretin (*TTR*), a transport protein for thyroxine and retinol produced mainly in the liver, and is classified as hereditary (ATTRv) or wild-type (ATTRwt). ATTRv is a severe, adult-onset systemic disease caused by pathogenic variants in the *TTR* gene and is transmitted in an autosomal dominant manner with incomplete penetrance (1).

Some pathogenic *TTR* variants are associated with a predominantly neurological phenotype of progressive sensorimotor polyneuropathy, whereas others are more strongly associated with restrictive cardiomyopathy; many produce a mixed phenotype. The advent of disease-modifying therapies, *TTR* tetramer stabilizers (tafamidis) and RNA-interference agents (patisiran, vutrisiran), has transformed ATTRv into a treatable disease, making early and definitive diagnosis critical (1, 2).

Although ATTRv shows geographic clustering in endemic foci, sporadic late-onset cases in non-endemic regions are increasingly reported (3, 4), heightening the importance of recognition in everyday practice. In Japan, two major endemic foci (Kumamoto and Nagano) are known; most early-onset cases occur in these foci, whereas late-onset cases are reported nationwide, often without an apparent family history.

In recent years ATTR has been emphasized as a cardiac disorder, presenting with ventricular hypertrophy, diastolic dysfunction, heart failure, and arrhythmia (5); consequently, cardiology-based screening of unexplained left ventricular hypertrophy is now widespread. By contrast, the neurological phenotype typically begins with small-fiber involvement, producing distal sensory disturbance, dysesthesia, and pain, with motor involvement appearing some years later (6). Because this picture resembles diabetic polyneuropathy and CIDP, diagnosis is frequently delayed, leading to delayed treatment and a worse prognosis.

Several prospective studies in Western countries have evaluated the prevalence of ATTRv by *TTR* sequencing in patients with idiopathic polyneuropathy. In a French nationwide prospective study, 2.7% of 553 patients older than 50 years with idiopathic polyneuropathy carried a pathogenic *TTR* variant (7). Likewise, a German study of 102 patients with idiopathic progressive polyneuropathy in non-endemic conurban areas identified amyloidogenic *TTR* variants in 2 patients (2.0%) (8). However, comparable screening studies in Japan are limited, and the prevalence and clinical features of ATTRv in Japanese patients—particularly in the neurology clinic—remain poorly defined.

Most patients with peripheral neuropathy attend a neurology clinic, but cardiac evaluation such as echocardiography is seldom performed at the first visit. Unless neurologists suspect ATTRv from neurological findings, diagnosis may be delayed before cardiac assessment is undertaken. Whereas cardiology has established hypertrophy-triggered diagnostic algorithms for ATTRv, no specific diagnostic flow exists for suspecting ATTRv in the neurology clinic before cardiac evaluation.

We therefore prospectively analyzed 130 consecutive patients evaluated for suspected peripheral neuropathy at our neurology clinic between 2020 and 2025. Comprehensive analysis of the *TTR* coding region was performed to determine the prevalence of ATTRv in a non-endemic region of Japan, to characterize the clinical features of the patients identified, and to describe neurological findings and findings on the ECG, an investigation readily available in any neurology clinic, that may inform future screening approaches.

## 2 Materials and methods

### 2.1 Study design and participants

This was a single-center prospective observational study. The St. Marianna Neurological Disease Registry (a registry study for the development of novel diagnostics, therapeutics, and preventive methods; UMIN000042192) enrolls patients with neurological disorders who provide written informed consent for the banking of biological samples and clinical data. Of 1,910 patients enrolled between 2020 and 2025, we included all 130 consecutive patients in whom peripheral neuropathy was suspected on clinical grounds at the time of enrollment, defined as the presence of symptoms or signs attributable to peripheral nerve dysfunction (distal sensory disturbance, distal weakness, or autonomic symptoms), irrespective of whether a working diagnosis had already been made. Patients with an established diagnosis such as CIDP or ALS were deliberately not excluded, because the aim of the study was to estimate the prevalence of ATTRv among all patients seen for peripheral nerve symptoms in a neurology clinic, and because ATTRv can mimic or coexist with these disorders; in ALS, for example, distal weakness with reduced compound muscle action potentials may resemble an axonal neuropathy in the early phase. Inclusion was thus based on clinical suspicion of peripheral neuropathy rather than on electrophysiological confirmation; nerve conduction studies were subsequently performed in 128 of the 130 patients (98.5%); an axonal and/or demyelinating abnormality was documented in 123 of these (96.1%), and the findings were normal in 5 (3.9%) (Table 1). Patients with normal conventional nerve conduction studies were retained because small-fiber or autonomic neuropathy, both characteristic of early ATTRv, may present with normal conventional nerve conduction studies. This inclusive definition should be taken into account when interpreting the reported prevalence (see Discussion); a sensitivity analysis excluding patients with ALS is also presented.

**Table 1:** Baseline characteristics of the study cohort (*N* = 130).

Variable	Value, % (*n*)
Demographics	
Male	56.2 (73)
Female	43.8 (57)
Age at onset, mean (years)	61.1
HbA1c ≥ 6.5%	12.3 (16)
Underlying diagnosis	
CIDP	27.7 (36)
ALS	13.8 (18)
Sjögren’s syndrome	4.6 (6)
Guillain–Barré syndrome	0.8 (1)
Diabetic neuropathy	0.8 (1)
Others	52.3 (68)
Neurological features	
Polyneuritis	68.5 (89)
Mononeuritis multiplex	12.3 (16)
Pain	33.8 (44)
Muscle weakness	33.8 (44)
Numbness	10.0 (13)
Autonomic symptoms	
Constipation	20.0 (26)
Dizziness	17.7 (23)
Response to immunotherapy	43.8 (57)
Cardiac—echocardiography	*N* = 97
Normal	44.3 (43)
Valvular heart disease	14.4 (14)
Other findings	55.7 (54)
Cardiac—electrocardiography	*N* = 98
Sinus rhythm	64.3 (63)
Arrhythmia	15.3 (15)
Low voltage	8.2 (8)
Other findings	10.2 (10)
Electrophysiology	*N* = 128
Axonopathy	46.1 (59)
Demyelination	37.5 (48)
Biopsy	*N* = 34
Amyloid deposition	8.8 (3)

CIDP, chronic inflammatory demyelinating polyradiculoneuropathy; ALS, amyotrophic lateral sclerosis; ECG, electrocardiography. Percentages for echocardiography, electrocardiography, electrophysiology, and biopsy are calculated within the examined subgroup (*N* shown); ECG and echocardiography were performed at the discretion of the attending neurologist and were not available for 32 and 33 patients, respectively.

Clinical data, nerve conduction studies, ECG, and echocardiography were obtained as part of routine clinical care at the discretion of the attending neurologist rather than according to a study-mandated protocol; consequently, ECG and echocardiography were not available for all patients, and the number of patients with available data is stated for each variable (see Results and Table 1). Time to diagnosis was defined as the interval from the onset of the first symptom considered attributable to ATTRv on retrospective review of the clinical records, to identification of the pathogenic *TTR* variant. Autonomic dysfunction was defined as the presence of orthostatic hypotension or syncope, urinary disturbance, or gastrointestinal symptoms (constipation or diarrhea) judged by the attending neurologist to be neurogenic.

### 2.2 Ethics and consent

This study was approved by the Ethics Committee of St. Marianna University School of Medicine (approval no. 4983) and was conducted in accordance with the Declaration of Helsinki. Written informed consent was obtained from all participants.

### 2.3 TTR genetic analysis

Genomic DNA was extracted from peripheral blood using a commercial kit (QIAamp DNA Blood, Qiagen). All four exons of the *TTR* gene, which together contain the complete coding region, were amplified by PCR using primers located in the flanking intronic sequences, so that each amplicon covered the exon together with its exon–intron boundaries and at least 50 bp of flanking intronic sequence. Amplicons were purified by ExoSAP treatment and sequenced bidirectionally with the forward and reverse primers by Sanger sequencing (SeqStudio, Applied Biosystems). Electropherograms were inspected manually; only base calls with a Phred quality score ≥ 20 were accepted, a variant was called only when it was present on both strands, and every variant was confirmed in an independent PCR and sequencing reaction. Sequences were compared with the reference sequence (NM_000371.4) to identify pathogenic variants. Variants are reported according to the current recommendations of the Human Genome Variation Society (HGVS), using the numbering of the full *TTR* precursor protein (e.g., p.(Val50Met)), together with the traditional amyloid nomenclature recommended by the International Society of Amyloidosis (ISA), which is based on the mature protein and omits the 20-residue signal peptide (e.g., ATTRV30M) (9). Detailed protocols for DNA extraction, PCR, and sequencing are provided in the Supplementary methods, and primer sequences are listed in Supplementary Table S1.

### 2.4 Statistical analysis

Continuous variables are summarized as means, because data for the variant-negative group were available only as group-level summary statistics (means); medians are not reported. Categorical variables are summarized as counts and percentages, with the number of patients for whom the variable was available as the denominator (this denominator is stated for each variable). The prevalence of ATTRv and all proportions are reported with exact (Clopper–Pearson) binomial 95% confidence intervals (CIs). Because only three patients were variant-positive, no hypothesis tests comparing the variant-positive and variant-negative groups were performed, as such comparisons lack statistical power; all between-group data are presented descriptively, and no correction for multiple comparisons was therefore applicable. The diagnostic performance of a low-voltage ECG for ATTRv (sensitivity, specificity, positive and negative predictive values, and likelihood ratios) was calculated among patients with available ECG data and is reported with exact 95% CIs; the 95% CI for the positive likelihood ratio was obtained by the log method after adding 0.5 to each cell of the 2 × 2 table because of the zero false-negative count. No odds ratio was estimated. These diagnostic-performance estimates rest on only three variant-positive patients, are highly imprecise, and are reported for descriptive, hypothesis-generating purposes only. In addition, the compound clinical–electrophysiological score of Tozza et al. (10) was calculated retrospectively from the clinical records and nerve conduction data obtained at the first evaluation. The score comprises seven items—motor symptoms (0–1), a history of carpal tunnel syndrome (0–1), and the amplitudes of the median and ulnar sensory action potentials and of the median, ulnar, and tibial CMAPs, each categorized as normal (0), reduced (1), or absent (2) according to our laboratory’s reference values—giving a total of 0–12, with a published cut-off of ≥ 5 (10). The score could be calculated for the 125 patients (all three variant-positive and 122 variant-negative patients) in whom all seven items were available; the proportion with a score ≥ 5 in each group and the corresponding diagnostic-performance estimates are reported descriptively with exact 95% CIs. Calculations were performed using GraphPad Prism software (version 8; GraphPad Software, San Diego, CA, United States).

## 3 Results

### 3.1 Cohort characteristics

The 130 patients comprised 73 men (56.2%) and 57 women (43.8%), with a mean age at onset of 61.1 years; 16 patients (12.3%) had HbA1c ≥ 6.5% (Table 1). The most frequent underlying diagnosis was CIDP (36 patients, 27.7%), followed by amyotrophic lateral sclerosis (18, 13.8%), Sjögren’s syndrome (6, 4.6%), and Guillain–Barré syndrome and diabetic neuropathy (1 each, 0.8%). Polyneuritis was present in 89 patients (68.5%) and mononeuritis multiplex in 16 (12.3%). Autonomic symptoms included constipation (26, 20.0%) and dizziness (23, 17.7%). Electrophysiological study was performed in 128 patients and showed axonopathy in 59 (46.1%) and demyelination in 48 (37.5%). An ECG was available for 98 patients (75.4%) and echocardiography for 97 (74.6%). These cardiac examinations were performed as part of routine clinical care at the discretion of the attending neurologist, mainly in patients admitted for diagnostic evaluation or in whom a systemic or cardiac disorder was clinically suspected; they were not obtained in 32 and 33 patients, respectively, most of whom were evaluated only as outpatients or already had an established alternative diagnosis. All three variant-positive patients underwent both examinations; ECG data were therefore available for 95 and echocardiography for 94 of the 127 variant-negative patients. A low-voltage ECG was recorded in 8 of the 98 patients with an ECG (8.2%). Biopsy was performed in 34 patients, and amyloid deposition was confirmed in 3 (8.8%).

### 3.2 Identification of TTR variants

Individual clinical features of the three patients are shown in Table 2. Three of the 130 patients (2.31%; exact 95% CI, 0.5–6.6%) carried a pathogenic *TTR* variant and were diagnosed with ATTRv: ATTRV122I [p.(Val142Ile)], ATTRI127V [p.(Ile147Val)], and ATTRV30M [p.(Val50Met)], all heterozygous. When the 18 patients with ALS were excluded, the prevalence was 3/112 (2.68%; 95% CI, 0.6–7.6%). None of the three had a family history of amyloidosis or neuropathy, and all had late-onset disease (age at onset 59–79 years; age at diagnosis 62–83 years), with an interval of 4–7 years between symptom onset and genetic diagnosis (Table 2). Two patients (Cases 1 and 2) had established progressive axonal or mixed polyneuropathy, whereas Case 3 had early, mild neuropathy in which prolonged distal latencies of the bilateral median nerves (a bilateral carpal-tunnel pattern) and reduced vibration sense were the only abnormalities. All three had autonomic dysfunction, gait instability, and a low or low-normal body mass index (18.0–21.2 kg/m^2^), and cardiac amyloid involvement was demonstrated in all three by echocardiography, 99mTc-PYP scintigraphy, or endomyocardial biopsy.

**Table 2:** Clinical features of the three patients diagnosed with ATTRv amyloidosis.

Variable	Case 1	Case 2	Case 3
Age/Sex	82/F	62/M	83/M
BMI (kg/m^2^)	19.0	18.0	21.2
Family history	None	None	None
Chief complaint	Distal numbness	Distal numbness	Dizziness/gait instability
Time to diagnosis (from first symptom onset)	7 years	4 years	4 years
Autonomic dysfunction	Urinary frequency	Severe orthostatic hypotension	Orthostatic hypotension
Gait instability	Present	Present	Present
Distal muscle weakness	Mild, asymmetric	Distal-dominant	Absent/minimal
Sensory disturbance	Glove-and-stocking dysesthesia	Distal sensory disturbance	Reduced vibration sense
NCS findings	Reduced CMAP, prolonged distal latency (at first visit, 2017)	Axonal, prolonged distal latency	Prolonged bilateral median distal latencies (carpal-tunnel pattern); no CMAP reduction
Electrophysiological pattern	Mixed/axonal	Predominantly axonal	Demyelinating-range distal latencies confined to the median nerves (bilateral CTS); early/mild
ECG	Low voltage	Low voltage	Low voltage, complete RBBB
Echocardiography (IVS, EF)	Concentric LVH; IVS 10 mm, EF 67%	Normal; IVS 9 mm, EF 71%	Diffuse LVH; IVS 15 mm, EF 73%
99mTc-PYP scintigraphy	Negative	Grade 2	Grade 3
Biopsy	Endomyocardial amyloid	Sural nerve: severe axonal loss	Cardiac amyloid
TTR variant	ATTRV122I [p.(Val142Ile)]	ATTRI127V [p.(Ile147Val)]	ATTRV30M [p.(Val50Met)]
Disease-modifying therapy	Vutrisiran	Under evaluation	Tafamidis → vutrisiran
Current ADL	Indoor assisted walking	Ambulatory	Independent

ATTRv, hereditary transthyretin amyloidosis; BMI, body mass index; CMAP, compound muscle action potential; CTS, carpal tunnel syndrome; ECG, electrocardiography; EF, ejection fraction; IVS, interventricular septum thickness; LVH, left ventricular hypertrophy; NCS, nerve conduction study; RBBB, right bundle branch block; 99mTc-PYP, 99mTc-pyrophosphate. TTR variants are given in the traditional amyloid (ISA, mature-protein) nomenclature with the current HGVS nomenclature based on the full precursor protein in square brackets. Time to diagnosis is the interval from the onset of the first symptom considered attributable to ATTRv on retrospective review, to identification of the pathogenic TTR variant. Case 3 was identified through a cardiology-initiated 99mTc-PYP scintigraphy pathway.

Descriptive comparisons between the variant-positive and variant-negative groups are shown in Table 3; because only three patients were variant-positive, no hypothesis tests were performed. The mean age at onset of the variant-positive patients was higher than that of the variant-negative group (71.0 vs. 60.9 years). A low-voltage ECG was present in all three ATTRv patients (3/3, 100%; 95% CI, 29.2–100%) and in 5 of the 95 variant-negative patients with an available ECG (5.3%; 95% CI, 1.7–11.9%). Autonomic dysfunction was present in 3/3 variant-positive patients (100%; 95% CI, 29.2–100%) and in 46/127 variant-negative patients (36.2%; 95% CI, 27.9–45.2%). Among the 98 patients with ECG data, the diagnostic performance of a low-voltage ECG for ATTRv was as follows: sensitivity 100% (3/3; 95% CI, 29.2–100%), specificity 94.7% (90/95; 95% CI, 88.1–98.3%), positive predictive value 37.5% (3/8; 95% CI, 8.5–75.5%), negative predictive value 100% (90/90; 95% CI, 96.0–100%), positive likelihood ratio 19.0 (continuity-corrected 95% CI, 6.3–37.3), and negative likelihood ratio 0 (not estimable). These estimates are highly imprecise because they rest on only three variant-positive patients. No pathogenic variant was identified among patients with pure demyelinating polyneuropathy lacking autonomic symptoms. The compound score of Tozza et al. (10) could be calculated for 125 patients. It was 5, 5, and 4 in Cases 1, 2, and 3, respectively, so that a score ≥ 5 was present in 2 of the 3 variant-positive patients (66.7%; 95% CI, 9.4–99.2%) and in 37 of the 122 variant-negative patients (30.3%; 95% CI, 22.3–39.3%) (Table 3). The corresponding sensitivity, specificity, positive predictive value, and negative predictive value of a score ≥ 5 for ATTRv in our cohort were 66.7% (2/3; 95% CI, 9.4–99.2%), 69.7% (85/122; 95% CI, 60.7–77.7%), 5.1% (2/39; 95% CI, 0.6–17.3%), and 98.8% (85/86; 95% CI, 93.7–100%), respectively. The patient with a score below the cut-off (Case 3) was the patient with early, mild neuropathy, whose ECG nevertheless showed low voltage.

**Table 3:** Descriptive comparison of clinical and cardiac findings between patients with and without a pathogenic *TTR* variant.

Variable	TTR variant present (*n* = 3)	TTR variant absent (*n* = 127)	95% CI (%), present/absent
Age at onset, mean (years)	71.0	60.9	—
Male sex, % (*n*/*N*)	66.7 (2/3)	55.9 (71/127)	9.4–99.2/46.8–64.7
HbA1c, mean (%)	6.6	5.8	—
ECG: low voltage, % (*n*/*N*)	100 (3/3)	5.3 (5/95)	29.2–100/1.7–11.9
Echocardiography: IVS, mean (mm)	11.3	8.6	—
Autonomic dysfunction, % (*n*/*N*)	100 (3/3)	36.2 (46/127)	29.2–100/27.9–45.2
Polyneuritis, % (*n*/*N*)	66.7 (2/3)	68.5 (87/127)	9.4–99.2/59.7–76.5
Mononeuritis multiplex, % (*n*/*N*)	33.3 (1/3)	11.8 (15/127)	0.8–90.6/6.8–18.7
Axonopathy, % (*n*/*N*)	33.3 (1/3)	46.4 (58/125)	0.8–90.6/37.4–55.5
Demyelination, % (*n*/*N*)	66.7 (2/3)	36.8 (46/125)	9.4–99.2/28.4–45.9
Compound score (Tozza) ≥ 5, % (*n*/*N*)	66.7 (2/3)	30.3 (37/122)	9.4–99.2/22.3–39.3

Data are descriptive; because only three patients were variant-positive, no hypothesis tests were performed. Percentages are calculated with the number of patients for whom the variable was available as the denominator (*N*): clinical variables, all 127 variant-negative patients; ECG, the 95 variant-negative patients with an ECG (all three variant-positive patients had an ECG); echocardiography, the 94 variant-negative patients with an echocardiogram; electrophysiology, the 125 variant-negative patients with a nerve conduction study; compound score, the 122 variant-negative patients in whom all seven score items were available. 95% CIs are exact (Clopper–Pearson) binomial intervals for the variant-present and variant-absent groups, respectively. Continuous variables are shown descriptively (mean) because per-patient raw data for the variant-absent group were summarized at the group level; no inferential statistic is given (—). The shaded row indicates the finding of principal interest (low-voltage ECG), which rests on three patients, is highly imprecise, and should be regarded as hypothesis-generating. The demyelination category for Case 3 reflects prolonged distal median-nerve latencies attributable to bilateral carpal tunnel syndrome. The compound score is the clinical–electrophysiological score of Tozza et al. (10) (range 0–12; published cut-off ≥ 5). ECG, electrocardiography; IVS, interventricular septum thickness; TTR, transthyretin.

### 3.3 Case presentations

#### 3.3.1 Case 1: ATTRV122I [p.(Val142Ile)]

An 82-year-old woman (height 139.3 cm, weight 36.9 kg, BMI 19.0 kg/m^2^) with diabetes mellitus presented with numbness of the limbs; there was no clear family history of neurological disease. She first presented in 2017 with a dropped head due to neck extensor weakness; nerve conduction studies performed at that first visit already showed reduced compound muscle action potentials (CMAPs) in the bilateral median and peroneal nerves and prolonged distal latency of the left peroneal nerve, although limb symptoms were absent. In retrospect, the dropped head was considered the first clinical manifestation potentially attributable to ATTRv. Lower-limb numbness appeared in 2020 and ascended to the upper limbs in 2021, with grip difficulty and gait instability. The pathogenic variant was identified in 2024, 7 years after the onset of the dropped head and 4 years after the onset of limb numbness. Examination showed mild, asymmetric weakness, glove-and-stocking dysesthesia, reduced lower-limb tendon reflexes, and urinary frequency. HbA1c was 8.3%; monoclonal protein and anti-acetylcholine-receptor antibody were negative. Although diabetes mellitus was present, the neuropathy was considered attributable predominantly to ATTRv rather than to diabetes, because of its ascent from the lower to the upper limbs within approximately one year with the development of motor deficits, the asymmetric motor involvement with reduced CMAPs that was already present in 2017, 3 years before the onset of limb numbness, the preceding axial (neck extensor) weakness that is not a feature of diabetic polyneuropathy, the accompanying weight loss and autonomic (urinary) symptoms, and the coexistence of biopsy-proven cardiac amyloidosis; a contribution of diabetic neuropathy cannot, however, be excluded. ECG showed low voltage, and echocardiography demonstrated concentric left ventricular hypertrophy, diffuse wall thickening, right ventricular hypertrophy, and an apical sparing pattern, suggesting cardiac amyloidosis; 99mTc-pyrophosphate (99mTc-PYP) scintigraphy was negative. *TTR* analysis identified a heterozygous p.(Val142Ile) variant, and endomyocardial biopsy confirmed amyloid deposition, establishing ATTRv. Vutrisiran was started after discharge.

#### 3.3.2 Case 2: ATTRI127V [p.(Ile147Val)]

A 62-year-old man (height 164.6 cm, weight 48.7 kg, BMI 18.0 kg/m^2^) with hypertension presented with limb numbness and approximately 10 kg of weight loss over 2 years; there was no notable family history. Numbness that began in 2022 worsened from April 2025, but spinal MRI and nerve conduction studies at another hospital were unremarkable and he was managed symptomatically. Subsequent distal weakness (difficulty opening bottles) prompted referral and admission in 2025; the pathogenic variant was identified in 2026, 4 years after symptom onset. Examination showed distal-dominant weakness, dysesthesia of the distal limbs, difficulty with tandem gait, a positive Romberg sign, and severe orthostatic hypotension with syncope; autonomic testing (CVR-R and head-up tilt) was positive. Nerve conduction studies showed mildly prolonged distal latencies of the bilateral median nerves and markedly reduced CMAP amplitudes in the bilateral peroneal and right tibial nerves, with reduced sensory nerve action potentials. Sural nerve biopsy showed marked loss of myelinated and unmyelinated fibers without demyelinating change. ECG showed low voltage; echocardiography was structurally unremarkable, but 99mTc-PYP scintigraphy showed Grade 2 uptake, suggesting cardiac amyloidosis. *TTR* analysis identified a heterozygous p.(Ile147Val) variant, establishing ATTRv. The neuropathy was attributed to ATTRv on the basis of the progressive axonal sensorimotor polyneuropathy with prominent autonomic failure and weight loss, the absence of diabetes or any other identifiable cause, and the demonstration of cardiac amyloid involvement by scintigraphy despite a structurally normal echocardiogram.

#### 3.3.3 Case 3: ATTRV30M [p.(Val50Met)]

An 83-year-old man (height 168.8 cm, weight 60.4 kg, BMI 21.2 kg/m^2^) with hypertension and old pulmonary tuberculosis developed recurrent vertigo with tinnitus and hyperacusis in 2021, accompanied by orthostatic hypotension and gait instability. Cardiac evaluation prompted by these symptoms included 99mTc-PYP scintigraphy, which showed Grade 3 uptake suggesting cardiac amyloidosis, and he was referred to our neurology clinic for assessment of neuropathy; the pathogenic variant was identified in 2025, 4 years after symptom onset. Thus, unlike Cases 1 and 2, this patient was identified through a cardiology-initiated scintigraphy pathway rather than through the neurology-based pathway proposed in this study. Neurological examination showed only reduced vibration sense in the left lower limb. Nerve conduction studies showed prolonged distal motor and sensory latencies of the bilateral median nerves (more marked on the left) without clear CMAP reduction; these findings were classified electrophysiologically as demyelinating-range abnormalities, but they were confined to the median nerves at the wrist and were interpreted as bilateral carpal tunnel syndrome, a recognized early manifestation of ATTRv, rather than as a diffuse demyelinating polyneuropathy. The neuropathy was therefore considered an early, mild sensory neuropathy with a bilateral carpal-tunnel pattern rather than an established axonal polyneuropathy, and its attribution to ATTRv was supported by the coexistence of orthostatic hypotension, bilateral carpal tunnel syndrome, biopsy-proven cardiac amyloidosis, and the pathogenic p.(Val50Met) variant, together with the absence of diabetes or another cause of neuropathy. ECG showed complete right bundle branch block and low voltage; echocardiography demonstrated diffuse ventricular wall thickening, reduced left ventricular longitudinal strain, right ventricular hypertrophy, and mitral and tricuspid valve thickening. *TTR* analysis identified a heterozygous p.(Val50Met) variant, establishing ATTRv. Tafamidis was started and later switched to vutrisiran; the patient remains independent in activities of daily living.

## 4 Discussion

In this prospective study of 130 consecutive patients evaluated for suspected peripheral neuropathy at a neurology clinic in a non-endemic region of Japan, 2.31% (3/130) carried a pathogenic *TTR* variant and were diagnosed with ATTRv. Prospective *TTR*-sequencing data on the prevalence of ATTRv among neuropathy patients in non-endemic Japan are scarce, and our study helps to define this. Beyond confirming the long-established association of ATTRv with progressive axonal polyneuropathy and autonomic dysfunction, the study yields four findings that we consider novel or of specific clinical value: (i) the prevalence of ATTRv among patients evaluated for peripheral neuropathy in a Japanese non-endemic neurology clinic (2.31%) is comparable to that reported in European cohorts; (ii) two of the three variants (ATTRV122I and ATTRI127V) were non-V30M variants unrelated to the Japanese endemic foci, and all three patients had late-onset disease without a family history; (iii) cardiac amyloid involvement was demonstrable in all three patients, including one whose echocardiogram was structurally normal and one whose neuropathy was still mild, and a low-voltage ECG was present in all three; and (iv) the uniform presence of a low-voltage ECG, which we report as a preliminary observation suggesting that a simple ECG, obtained in parallel with genetic testing, may provide a complementary clue to occult cardiac involvement in the neurology clinic, where echocardiography is not routinely performed.

ATTRv is an autosomal dominant disease in which amyloid deposits in peripheral and autonomic nerves, the heart, kidneys, gastrointestinal tract, and eyes, producing multi-organ dysfunction; untreated, the mean interval from onset to death is approximately 7–10 years (11, 12). In endemic foci, early-onset cases are readily suspected from family history and characteristic features, but late-onset cases in non-endemic regions, with heterogeneous presentations, late onset, and absent family history, are prone to delayed or mistaken diagnosis (13, 14). Because disease-modifying therapies can slow progression, early diagnosis is increasingly important.

ATTRv is difficult to diagnose early because its features are diverse and non-specific, onset spans the third to eighth decades, and family history is often absent (15). Traditionally, when red-flag findings raise suspicion, diagnosis is confirmed by histological demonstration of amyloid followed by *TTR* sequencing (16, 17). Internationally, efforts to identify ATTRv earlier among patients with peripheral neuropathy are emerging (2, 18, 19); for example, a UK and Ireland expert consensus recommends prompt *TTR* testing when red-flag findings accompany peripheral neuropathy (19). Because amyloid detection on biopsy varies by site and is not uniform, a negative biopsy does not exclude amyloidosis (16, 20). Accordingly, Maruyama et al. proposed a flow permitting *TTR* testing before histology when suspicion is high or biopsy is difficult, while retaining both variant identification and tissue confirmation (including bone scintigraphy) for a definitive diagnosis given incomplete penetrance (20).

ATTRV122I [p.(Val142Ile)] is relatively common in people of African and Asian descent, and heterozygotes typically develop late-onset cardiac amyloidosis (21, 22); Case 1, with cardiac-predominant disease and mild neuropathy, was consistent with this, although her initial manifestation was a dropped head, an unusual presentation that preceded limb symptoms by 3 years. ATTRI127V [p.(Ile147Val)] has been reported to involve the glossopharyngeal, vagal, and hypoglossal nerves, causing dysphagia and respiratory complications (23); Case 2 had no overt cardiac lesion but showed distal-dominant weakness and orthostatic hypotension, suggesting a progressive course. Notably, the same p.(Ile147Val) variant was among the amyloidogenic variants identified in the German idiopathic-neuropathy cohort, supporting its relevance to neuropathy-predominant ATTRv in non-endemic regions (8). ATTRV30M [p.(Val50Met)], the classic variant of the Japanese endemic foci, involves sensory, motor, and autonomic nerves, with autonomic dysfunction prominent in early-onset disease (24); Case 3 showed diffuse left ventricular wall thickening, reduced vibration sense with a bilateral carpal-tunnel pattern, and orthostatic hypotension, combining early peripheral, autonomic, and cardiac involvement. Thus, all three cases broadly matched the known phenotypes, but their identification in a non-endemic region and their phenotypic diversity carry implications for diagnostic strategy. In the international THAOS registry, late-onset ATTRV30M was frequent and already more severe at enrolment, underscoring that heightened awareness may enable earlier diagnosis (25). Case 3 is also consistent with the natural history of late-onset ATTRV30M in non-endemic areas of Japan described by Koike et al., in which onset typically occurs after the age of 50 years, a family history is frequently absent, sensory loss involves all modalities, autonomic symptoms are relatively mild, cardiac involvement is common, and neurological disability progresses steadily over several years (26). In this context, the mild neurological findings of Case 3 are best interpreted as an early stage of a progressive disease—captured because cardiac amyloidosis had already been recognized—rather than as a benign phenotype, which underscores the value of early disease-modifying therapy.

The finding of a low-voltage ECG in all three patients deserves comment, because low QRS voltage is reported in only about a quarter of patients with ATTRv cardiomyopathy in cardiology series and has been regarded as a relatively late finding (27, 28); its presence in all three of our patients is therefore likely to reflect, at least in part, the small sample. In Cases 1 and 3, low voltage is readily explained by established cardiac amyloid involvement (concentric left ventricular hypertrophy with an apical sparing pattern in Case 1, and biopsy-proven cardiac amyloidosis with diffuse wall thickening in Case 3), and in both the combination of low voltage with increased wall thickness—the voltage-to-mass discordance characteristic of cardiac amyloidosis—was present. Case 2 (ATTRI127V), who had low voltage despite a structurally unremarkable echocardiogram, is more difficult to interpret. We consider the most likely explanation to be early myocardial amyloid infiltration that had not yet produced measurable wall thickening: Grade 2 99mTc-PYP uptake, which in the absence of a monoclonal protein is diagnostic of cardiac ATTR amyloidosis (29), documented myocardial amyloid deposition, whereas the patient’s low BMI (18.0 kg/m^2^) and the absence of pericardial effusion argue against a habitus or effusion-related reduction in voltage. An alternative interpretation is that the ECG and echocardiographic findings were discordant by chance. Because cardiac magnetic resonance and serial strain echocardiography were not performed, the discrepancy cannot be resolved with the available data. Nevertheless, the observation that scintigraphy and the ECG were abnormal while the echocardiogram was normal is consistent with the recognized capacity of bone scintigraphy to detect cardiac ATTR amyloid at an early stage (29), and cautions against relying on echocardiography alone to exclude cardiac involvement in neuropathy-predominant ATTRv.

Cases 1 and 2 shared low body weight, gait instability, and distal-dominant weakness with muscle atrophy, and all three patients had gait instability and autonomic dysfunction. Gait instability in particular suggests impaired deep sensation. Capturing these features at the first outpatient visit may enable earlier detection of mild or atypical ATTRv that would otherwise be overlooked, allowing earlier treatment and preservation of daily function.

Two of the three diagnosed patients had progressive axonal or mixed polyneuropathy, the third had early, mild neuropathy with a bilateral carpal-tunnel pattern, and all three had autonomic dysfunction, whereas none of the patients with pure demyelinating polyneuropathy lacking autonomic symptoms carried a variant, consistent with ATTRv being primarily an axonal disorder. Importantly, cardiac amyloid involvement was demonstrable in all three patients even though cardiac symptoms were not overt at neurological presentation, and in Case 2 the echocardiogram was structurally normal although the ECG showed low voltage and scintigraphy was positive. Cardiology-led, hypertrophy-triggered screening alone may therefore miss such early cases, whereas in the neurology clinic, where echocardiography is seldom performed at the first visit, a 12-lead ECG is inexpensive and universally available. In patients with unexplained progressive peripheral neuropathy, established red flags (autonomic dysfunction, gait instability, low body weight or weight loss, distal-dominant weakness, severe neuropathic pain, prolonged distal latency on nerve conduction studies, bilateral carpal tunnel syndrome, or progression unresponsive to immunotherapy or unexplained by diabetes) should raise suspicion of ATTRv (2, 15, 16, 19). When such suspicion arises, *TTR* genetic testing—accompanied by appropriate genetic counseling—is the definitive first-line test and should be placed at the top of the diagnostic work-up rather than deferred until cardiac investigations have been completed; our finding that pathogenic variants were present in 2.31% of an unselected neurology-clinic cohort, together with the availability of disease-modifying therapy, supports a low threshold for requesting it. Our observation that a low-voltage ECG was present in all three ATTRv patients but in only 5.3% (5/95) of variant-negative patients with an available ECG raises the possibility that a 12-lead ECG obtained in parallel at that point could provide an additional, inexpensive clue to otherwise occult systemic (cardiac) involvement, reinforcing the indication for genetic testing and prompting cardiac assessment. We wish to be explicit that this is a preliminary observation and not evidence for a validated screening strategy, and that the ECG is proposed as a complement to, not a prerequisite for, genetic testing. The observation was made in the same three patients whose data are used to describe it; one of them (Case 3) was identified through a cardiology-initiated scintigraphy pathway and therefore does not bear on a neurology-based pathway at all; the diagnostic-performance estimates are highly imprecise (sensitivity 100%, 95% CI 29.2–100%); and because low QRS voltage is present in only a minority of patients with ATTRv cardiomyopathy in cardiology series (27, 28), a normal ECG must not be used to exclude ATTRv when neurological red flags are present. Whether the ECG adds to the detection of ATTRv in the neurology clinic can only be established in larger, independent, prospective cohorts; an illustrative framework in which *TTR* genetic testing and ECG/cardiac assessment are performed in parallel once red flags are present is provided as Supplementary Figure S1 for that purpose only, not as a recommended algorithm.

A complementary, purely neurological approach to selecting patients for *TTR* testing is the compound clinical–electrophysiological score of Tozza et al., which combines motor symptoms, a history of carpal tunnel syndrome, and categorized amplitudes of the median and ulnar sensory action potentials and of the median, ulnar, and tibial CMAPs (range 0–12); in the original study a score ≥ 5 discriminated ATTRv from chronic idiopathic axonal polyneuropathy with a sensitivity of 96.6% and a specificity of 63.6% (10). Applied retrospectively to the 125 patients of our cohort with complete data, a score ≥ 5 was present in 2 of the 3 variant-positive patients (scores 5, 5, and 4) and in 30.3% (37/122) of variant-negative patients. The proportion of variant-negative patients above the cut-off is similar to the 36.4% reported for chronic idiopathic axonal polyneuropathy in the original study (10), indicating that the score behaves similarly in an unselected neurology-clinic population that includes inflammatory, motor neuron, and other neuropathies, and that it would have flagged roughly one-third of our cohort for *TTR* testing. Two observations deserve emphasis, with the caveat that they rest on three patients. First, the patient below the cut-off (Case 3, score 4) was the patient with early, mild neuropathy, whose only neurophysiological abnormalities were prolonged distal median-nerve latencies; because the score weights axonal loss in five nerves and motor involvement, it is expected to be least sensitive at precisely this earliest stage, whereas the ECG of this patient already showed low voltage. Second, the two patients above the cut-off were also those with a low-voltage ECG, so the two approaches were concordant in the patients with established neuropathy. Because the compound score requires no cardiac investigation, whereas the ECG is a cardiac test that is nevertheless available in every neurology clinic, the two approaches appear complementary rather than competing; a prospective study applying both the compound score and a 12-lead ECG to consecutive patients with peripheral neuropathy would be a natural next step, and our registry provides a platform for such a study. These retrospective results should not be regarded as a validation or a refutation of the compound score, which was derived in a different setting and against a different comparison group.

Beyond clinical red flags, electrophysiological scores, and cardiac evaluation, emerging biomarkers may contribute to earlier recognition and monitoring of ATTRv. Neurofilament light chain (NfL), a marker of neuroaxonal injury, is elevated in the plasma of patients with ATTRv polyneuropathy, correlates with the severity of neuropathy, and has been proposed as an early marker of disease onset in variant carriers and as a marker of treatment response (30, 31). At present, therefore, early diagnosis rests on three complementary pillars: genetic testing with appropriate genetic counseling, which remains fundamental for identifying pathogenic *TTR* variants in patients and at-risk relatives; emerging biomarkers such as NfL; and careful longitudinal clinical assessment of early neurological manifestations in patients and in asymptomatic carriers. NfL and other biomarkers were not measured in the present cohort. Future prospective studies integrating genetic testing, biomarkers such as NfL, and longitudinal clinical assessment may improve the detection of disease onset in *TTR* variant carriers, and our registry, which banks biological samples, provides a platform for such work.

In the French nationwide prospective study, 2.7% of 553 patients older than 50 years with idiopathic polyneuropathy carried a pathogenic *TTR* variant (7). Our prevalence of 2.31% (2.68% after excluding patients with ALS) is broadly consistent with both this study and the German cohort (2.0%) (8), indicating that a meaningful proportion of ATTRv cases may be latent even in non-endemic Japan.

This study has several limitations. First, it was a single-center prospective observational study restricted to registry enrollees, and the inclusive eligibility criteria—patients with established diagnoses such as CIDP or ALS were not excluded—may have diluted the prevalence estimate; excluding the 18 patients with ALS yielded a prevalence of 2.68% (3/112). Second, only three patients were variant-positive; accordingly, no hypothesis tests were performed, all between-group data are descriptive, the exact 95% CIs are wide, and the ECG observation is preliminary and hypothesis-generating; the diagnostic performance and clinical utility of the ECG as a complementary clue cannot be established from these data. Third, ECG and echocardiography were obtained at the discretion of the attending neurologist rather than by protocol and were missing in 32 (24.6%) and 33 (25.4%) patients, respectively, mainly among patients evaluated only as outpatients or in whom an alternative diagnosis had been established. Because patients with a higher clinical suspicion of a systemic or cardiac disorder were more likely to be examined, the frequency of low-voltage ECG among variant-negative patients with available data may not represent that of the whole cohort, and the specificity of a low-voltage ECG may have been either over or underestimated; the direction of this selection bias cannot be determined from our data. Fourth, Case 3 was identified through a cardiology-initiated scintigraphy pathway rather than through a neurology-based pathway. Fifth, Sanger sequencing cannot detect copy-number or deep-intronic variants. Sixth, the compound score of Tozza et al. (10) was calculated retrospectively from data collected for clinical purposes, in 125 of the 130 patients, and in a cohort that differs from the one in which the score was derived; its performance here is descriptive only. Seventh, penetrance cannot be estimated from this cohort: the study enrolled patients evaluated for suspected peripheral neuropathy rather than unselected *TTR* variant carriers or family members, and all three carriers identified were symptomatic, so the asymptomatic-carrier denominator and the longitudinal family data required for a meaningful estimate of age-dependent penetrance are lacking; prospective follow-up of asymptomatic carriers identified through cascade testing is an important subject for future study. Despite these limitations, the study provides real-world prevalence data from a neurology clinic in non-endemic Japan supports early *TTR* genetic testing in patients with unexplained progressive neuropathy and red flags, and offers a preliminary observation—a low-voltage ECG in all three variant-positive patients—that merits prospective testing as a complementary clue.

In a non-endemic region of Japan, a pathogenic *TTR* variant was identified prospectively in 2.31% (3/130) of patients evaluated for suspected peripheral neuropathy, a frequency comparable to that in European cohorts, and two of the three variants were non-V30M variants unrelated to the Japanese endemic foci. All three patients had late-onset disease without a family history, showed autonomic dysfunction and gait instability, and had cardiac amyloid involvement with a low-voltage ECG even when neurological or echocardiographic findings were mild. These findings support placing *TTR* genetic testing, with genetic counseling, at the top of the diagnostic work-up in patients with unexplained progressive neuropathy and red flags, even in non-endemic regions. The uniform presence of a low-voltage ECG is a preliminary, hypothesis-generating observation suggesting that a simple ECG obtained in parallel may provide a complementary clue to occult cardiac involvement; this must be established in larger, independent, prospective cohorts.

## Data Availability

The original contributions presented in the study are included in the article/Supplementary material, further inquiries can be directed to the corresponding author.

## Glossary

ADL: Activities of daily living
ALS: Amyotrophic lateral sclerosis
ATTR: Transthyretin amyloidosis
ATTRv: Hereditary (variant) transthyretin amyloidosis
ATTRwt: Wild-type transthyretin amyloidosis
BMI: Body mass index
CIDP: Chronic inflammatory demyelinating polyradiculoneuropathy
CI: Confidence interval
CMAP: Compound muscle action potential
CTS: Carpal tunnel syndrome
CVR-R: Coefficient of variation of R-R intervals
ECG: Electrocardiography
EF: Ejection fraction
HbA1c: Hemoglobin A1c
HGVS: Human Genome Variation Society
ISA: International Society of Amyloidosis
IVS: Interventricular septum thickness
LR: Likelihood ratio
LVH: Left ventricular hypertrophy
MRI: Magnetic resonance imaging
NCS: Nerve conduction study
NPV: Negative predictive value
PCR: Polymerase chain reaction
PPV: Positive predictive value
RBBB: Right bundle branch block
RNA: Ribonucleic acid
TTR: Transthyretin
99mTc-PYP: Technetium-99 m pyrophosphate
