# Supplementary material for: Hereditary transthyretin amyloidosis in peripheral neuropathy: prevalence, clinical features, and neurology-based screening in non-endemic Japan
Source: Front Neurol. 2026 Oct 22;17:1955913. doi: 10.3389/fneur.2026.1955913 (PMC13735610; doi:10.3389/fneur.2026.1955913)
Supplement: Supplementary file 1 [file Supplementary_file_1.docx]

**Supplementary Methods**

Detailed protocols for genomic DNA extraction, PCR amplification, and Sanger sequencing of the *TTR* gene. Reagent volumes and cycling conditions are provided for reproducibility; primer sequences are listed in Supplementary Table 1.

**1. Blood collection**

Whole blood was collected into commercially available EDTA-treated tubes, gently mixed, and stored at room temperature until processing.

**2. Genomic DNA extraction**

Genomic DNA was extracted using the QIAamp DNA Blood Maxi Kit (Qiagen, Hilden, Germany) according to the manufacturer's instructions. In brief:

1) Two 7-mL blood samples were centrifuged at 3,000 rpm for 10 min; plasma was removed, and the sample was transferred to a 50-mL tube and adjusted to 10 mL with PBS.
2) QIAGEN Protease (500 µL) and RNase A (200 µL) were added with mixing.
3) Buffer AL (12 mL) was added; the tube was inverted 15 times, vortexed vigorously for ≥1 min, and incubated at 70 °C for 10 min.
4) After cooling to room temperature, ethanol (99.5%, 10 mL) was added; the tube was inverted 10 times and vortexed.
5) Half of the lysate was applied to a QIAamp Maxi column and centrifuged at 3,000 rpm for 5 min; the filtrate was discarded and the remaining lysate applied and centrifuged again.
6) The column was washed with Buffer AW1 (5 mL; 4,400 rpm, 5 min) and Buffer AW2 (5 mL; 4,400 rpm, 15 min).
7) DNA was eluted with 1,000 µL of room-temperature purified water applied directly onto the membrane, incubated 5 min, and centrifuged at 4,400 rpm for 5 min.

**3. DNA quantification**

DNA concentration and purity were assessed with a NanoDrop One spectrophotometer (A260/A280 ratio), using purified water as the blank.

**4. PCR amplification**

Exons 1–4 of the *TTR* gene, which together contain the complete coding region, were amplified using exon-specific forward and reverse primers located in the flanking intronic sequences so that each amplicon covered the exon together with its exon–intron boundaries. Primer sequences are listed in Supplementary Table 1~~.~~ Each 10-µL reaction contained 5 µL PCR Master Mix (Promega, Madison, WI, USA), 1 µL forward primer (10 µM), 1 µL reverse primer (10 µM), 2 µL nuclease-free water, and 1 µL template DNA. Cycling conditions: initial denaturation at 95 °C for 2 min; 35 cycles of 95 °C for 30 s (denaturation), 60 °C for 30 s (annealing), and 72 °C for 1 min (extension); final extension at 72 °C for 5 min; hold at 4 °C. Amplification was confirmed by electrophoresis on a 1% agarose gel (1× TAE buffer).

**5. Purification and sequencing**

PCR products were purified by ExoSAP treatment: 1.6 µL ExoSAP-IT™ PCR Product Cleanup Reagent (Applied Biosystems/Thermo Fisher Scientific, Waltham, MA, USA) was added to 5 µL PCR product, incubated at 37 °C for 4 min, and inactivated at 80 °C for 1 min. Each amplicon was sequenced bidirectionally in two separate reactions, one with the forward and one with the reverse PCR primer. Sequencing reactions (21 µL) contained 18 µL nuclease-free water, 1 µL primer, and 2 µL purified DNA, and were analysed on an automated sequencer (SeqStudio Genetics Analyzer, Applied Biosystems/Thermo Fisher Scientific, Waltham, MA, USA).

**6. Variant identification**

Sequences were compared with the *TTR* reference sequence (GenBank NM_000371.4); alignments and electropherograms were inspected manually to identify pathogenic variants. Only base calls with a Phred quality score ≥ 20 were accepted; a variant was called only when it was present on both the forward and the reverse strand, and every variant was confirmed in an independent PCR and sequencing reaction. Variants are reported according to the current HGVS recommendations using the numbering of the full *TTR* precursor protein (e.g., p.(Val50Met)), together with the traditional amyloid nomenclature recommended by the International Society of Amyloidosis (ISA), which is based on the mature protein and omits the 20-residue signal peptide (e.g., ATTRV30M).
